# Challenges in Cytology Specimens With Hürthle Cells

**DOI:** 10.3389/fendo.2021.701877

**Published:** 2021-06-25

**Authors:** Eleni Thodou, Sule Canberk, Fernando Schmitt

**Affiliations:** ^1^ Department of Pathology, Medical School, University of Thessaly, Larissa, Greece; ^2^ Instituto de Investigação e Inovação em Saúde (i3S), University of Porto, Porto, Portugal; ^3^ Institute of Molecular Pathology and Immunology, University of Porto (Ipatimup), Porto, Portugal; ^4^ Medical Faculty, Porto University, Porto, Portugal; ^5^ CINTESIS@RISE, Porto, Portugal

**Keywords:** Hürthle cell tumors, thyroid FNA, cytology, oncocytic tumors, neoplasia

## Abstract

In fine-needle aspirations (FNA) of thyroid, Hürthle cells can be found in a broad spectrum of lesions, ranging from non-neoplastic conditions to aggressive malignant tumors. Recognize them morphologically, frequently represents a challenging for an adequately diagnosis and are associated with a significant interobserver variability. Although the limitations of the morphologic diagnosis still exist, the interpretation of the context where the cells appear and the recent advances in the molecular knowledge of Hürthle cells tumors are contributing for a more precise diagnosis. This review aims to describe the cytology aspects of all Hürthle cells neoplastic and non-neoplastic thyroid lesions, focusing on the differential diagnosis and reporting according to The Bethesda System for Reporting Thyroid Cytology (TBSRTC). New entities according to the latest World Health Organization (WHO) classification are included, as well as an update of the current molecular data.

## Introduction

The term “Oncocyte/Hürthle cell” indicates the particular morphological appearance of a thyrocyte which has a “swollen” cytoplasm due to the abundant increase in the amount of abnormally dysfunctional mitochondria. The cells are characterized by a large cytoplasm displaying a dense, granular, eosinophilic color with a distinct cell border and pink macronucleoli (1). What is defined by pathologists as “Oncocyte/Hürthle” and “oncocytoid/mitochondrion-rich” cells are a reflection of morphological alterations based on the number of cationic organelles in the cell by showing a spectrum from fully, dense, pink cytoplasm (oncocyte/Hürthle) to the less or incomplete oncocytic appearance (“oncocytoid/mitochondrion-rich” cells). However, neither at microscopy, nor at ultrastructural level, exists quantitative measurements for an exact distinction between “oncocytoid” cells from non-oncocytic counterpart (2).

Oncocytic cells are present in many benign and malignant lesions in all endocrine organs. They have been originally associated with senescence. They are encountered in chronic inflammatory lesions as a way of cell adaptation to stress. Oncocytic cell phenotype is considered a result of metaplasia. However, the oncocytic cell is not another distinct cell type. It rather represents a different phenotype of the same cell as an adaptation phenomenon related to changed mitochondrial homeostasis. Regarding the thyroid both follicular and C cells may show oncocytic profile. In addition, oncocytic cells express the same immunohistochemical markers with the cells of origin (3).

In thyroid, oncocytic cells are observed as focal areas in nodular goiter or forming hyperplastic adenomatoid nodules. They are also encountered in autoimmune disorders as chronic lymphocytic thyroiditis (CLT) and in nodules arising in its background, in long-standing Graves’ disease, after head and neck irradiation therapy, and systematic chemotherapy (4).

In the 4th edition of the World Health Organization (WHO) Classification of Tumours of Endocrine Organs, Hürthle cell tumors (HCT) were accepted as a clinic-pathological entity that encompass benign and malignant neoplasms and not anymore variants of follicular adenoma and follicular carcinoma (5). Together with the new classification, one has kept the presence of oncocytic variants of papillary/medullary/poorly differentiated carcinoma (5). Recently, even cases of non-invasive follicular thyroid neoplasm with papillary like nuclear features (NIFTP) showing oncocytic phenotype, have been described (6–8).

In thyroid cytology the discrimination between a true HCT and other thyroid tumours with oncocytic features is not always feasible in routine practice (9). During the past decade, molecular tests on fine needle aspiration (FNA) specimens have provided a deeper inside in the pathogenesis of Hürthle cells neoplasms (HCN), yet their accurate preoperative diagnosis is still a challenge (10).

Oncocytic lesions in thyroid cytology befall to almost all categories of the Bethesda system for reporting thyroid cytopathology (TBSRTC) (11). In routine practice, lesions with oncocytic cells are reported with higher frequency in the indeterminate categories, namely atypia of undetermined significance/follicular lesion of undetermined significance (AUS/FLUS) and suspicious for follicular neoplasm/follicular neoplasm (SFN/FN), comparing to non-oncocytic lesions. They are also a source of increased interobserver variability (12, 13).

In this review comprehensive cytology of all Hürthle cells neoplastic and non-neoplastic thyroid lesions is described, focusing on the differential diagnosis and reporting according to TBSRTC. New entities according to the latest WHO classification are included, not reported in recent reviews and an update of the current molecular data is attempted.

## Non-Neoplastic Conditions With Hürthle Cell Morphology

### Chronic Lymphocytic Thyroiditis (CLT)

The most frequent lesion showing oncocytic cells in FNA smears is CLT. The cytology depends on the stage of the disease. In early stages, the samples are hypercellular composed of oncocytic follicular cells admixed with polymorphic lymphoid population. In later stages, fibrosis may be the reason for hypocellular samples. The typical cytology of Hashimoto thyroiditis shows oxyphilic cells arranged in usually small monolayered “honeycomb” groups, or isolated. They have abundant granular cytoplasm with well-defined borders and enlarged, hyperchromatic, often pleomorphic pyknotic nuclei, with conspicuous nucleoli. The lymphoid component consists of small mature lymphocytes, plasma cells, and germinal center cells and lympho-histiocytic aggregates (4, 14).

It is well established that CLT is a major source of false-positive in thyroid FNAs (15–17). Occasionally, focal reparative atypia of Hürthle cells in CLT can be observed. It usually presents as enlarged nuclei, with delicate smudgy chromatin, anisonucleosis, conspicuous nuclear membranes, macronucleoli, nuclear grooves, and even rare nuclear pseudoinclusions and may pose difficulties in differentiating from papillary thyroid carcinoma (PTC) (18–20). If this cytological atypia is not predominant, not associated with other features of PTC, but focal in a background of CLT, it is more appropriate to classify the lesion as AUS/FLUS, instead of suspicious for malignancy (SFM) (11).

Dominant hyperplastic Hürthle cell nodules in CLT may be a diagnostic challenge in FNA. The specimen may consist exclusively of oxyphilic cells with few or absent lymphocytes. The smears can be cellular showing macrofollicular, microfollicular, trabecular, or solid arrangement of epithelial cells with different amounts of colloid present. In addition, if Hürthle cells have prominent nucleoli and intervening blood vessels are present, the risk of misinterpreting the lesion for a HCN is high (19, 20).

According to an earlier publication, the number of lymphocytes as a single feature is not enough to differentiate CLT from a thyroid neoplasm (21). However, the presence of lymphocytes, especially percolating the cell groups, strongly suggests a nonneoplastic Hürthle cell nodule (4). Furthermore, clinical parameters as ultrasound features and serological tests for antithyroglobulin and anti-thyroid peroxidase antibodies should be taken into consideration in such cases.

If the clinical setting is suggestive of Hashimoto thyroiditis it is prudent not to sign out the case as follicular neoplasm Hürthle cell type/suspicious for follicular neoplasm Hürthle cell type (FNHCT/SFNHCT), but rather as category AUS/FLUS, according to TBSRTC. An explanatory note should be provided that a sample consisting of Hürthle cells in a patient with Hashimoto thyroiditis, more likely represents a hyperplastic nodule; however, a Hürthle cell neoplasm cannot be entirely excluded (11).

### Multinodular Goiter (MNG)

Nodules in MNG show diverse architectural histological patterns. Commonly, hyperplastic nodules consist of macrofollicles and/or microfollicles, occasionally showing oxyphilic changes to a variable extent. Atypia of the oncocytic cells similar to that seen in CLT such as anisonucleosis, prominent nucleoli, and multinucleation can also focally be found in MNG and Graves’ disease (21).

These histological features are reflected in FNA specimens. In a setting of a benign hyperplastic nodule, oncocytic cells may be present in various numbers forming sheets or isolated and occasionally showing significant anisonucleosis and hyperchromasia. In addition, transitional cell forms from regular follicular cells to oncocytic cells may be noted (14). This admixture of cell types is consistent with a hyperplastic nodule and should be interpreted as benign, according to TBSRTC (11).

However, when a hyperplastic nodule in MNG is composed predominantly of oxyphilic cells, the issue of differentiating from a HCN emerges. Important parameters to consider are the presence of colloid, inflammatory cells, namely lymphocytes and histiocytes, and the percentage of oncocytic cells, in order to avoid overcalling a hyperplastic nodule as neoplasm. Hypercellular samples with a monotonous Hürthle cell population of more than 90% of the specimen is a reliable criterion favoring neoplasia (4). Furthermore, a non-macrofollicular cell arrangement, presence of transgressing vessels and prominent macronucleoli are common features in neoplasms. Nevertheless, a false positive diagnosis cannot always be avoided (22–25).

In patients with multiple nodules, an FNA sample composed exclusively of Hürthle cells can be either classified as FNHCT/SFNHCT or as AUS/FLUS. In the latter case, an explanatory note defining that in the setting of MNG, it probably represents a hyperplastic nodule; nevertheless, a neoplastic process cannot be excluded. The AUS/FLUS category is meant to give the clinician the chance to decide towards a sonographic and clinical follow up rather than surgery (11).

Reparative changes are encountered in nodular goiter, particularly in nodules with cystic degeneration. Atypical cyst lining cells are elongated cells arranged in monolayers with well-defined cell borders, enlarged oblong nuclei and distinct nucleoli, occasionally resembling oncocytic cells. They should not be confused with Hürthle cells, because their cytoplasm is not dense and granular as in oxyphilic cells but rather delicate basophilic. In addition, they may have grooves, and rarely scant intranuclear inclusions. These cells, often representing a small portion of the follicular cell population, are usually easily recognized (19, 26). When such changes are more widespread than focal in the specimen may raise a concern for papillary carcinoma. If this is the case it should be classified as AUS/FLUS (11).

## Neoplastic Conditions With Hürthle Cell Morphology

### Hürthle Cell (ONCOCYTIC) Neoplasms (HCN)

**Figure 1:**
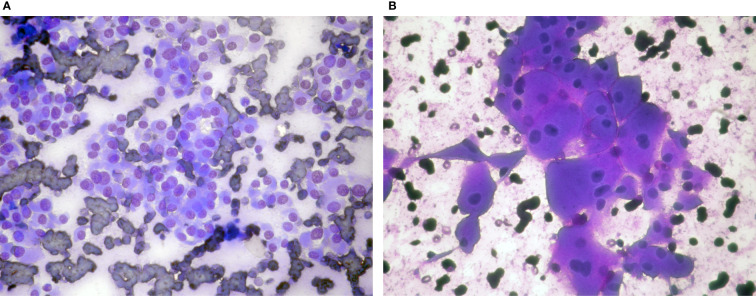
Hürthle cell neoplasms: **(A)** (Giemsa stain ×40) Microfollicles and dyscohesive cells with small cell dysplasia, **(B)** (Giemsa stain ×40) Monolayer of cells with large cell dysplasia. Note binucleation and prominent nucleoli.

**Figure 2:**
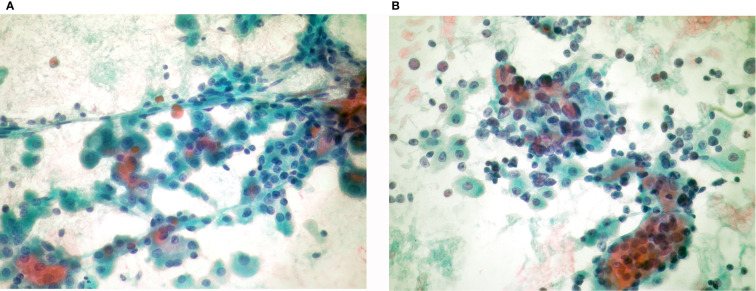
Hürthle cell neoplasms: **(A)** (Pap stain ×40) Hürthle cell adenoma: Monolayered groups and microfollicles with intervening blood vessels. **(B)** (Pap stain ×40) Hürthle cell carcinoma: Syncytial, three dimensional crowded cell groups, dispersed cells, naked nuclei, marked anisonucleosis, hyperchromasia, and cherry red macronucleoli.

Hürthle cell (oncocytic) tumors are usually encapsulated neoplasms, consisting of follicular cells with oxyphilic morphology in at least 75% of their cell population (5). Two types of tumors are included in this category: adenoma (HCA) and carcinoma (HCC). Discrimination of these two entities is strictly defined by histology, based on capsular and/or vascular invasion. The criteria for diagnosing a HCN in FNA include cellular smears with monotonous, predominantly (>90%) oncocytic cell population and absence of lymphocytes and plasmacytes (4, 23–25). Colloid is usually absent or scant (22, 23). However, cases of HCAs and HCCs showing abundant colloid have been reported (27). The cells are arranged in crowded microfollicular, syncytial, or trabecular aggregates or may be dyscohesive. They have enlarged central or eccentric round nucleus with macronucleoli and commonly show binucleation (22, 25). In addition, “small” or “large cell dysplasia” has been described as a characteristic of neoplastic Hürthle cells, initially identified in cases of HCC (28, 29). In “small cell dysplasia” the cell diameter is less than twice the nuclear diameter, which means cells smaller than the usual Hürthle cells with a higher nuclear cytoplasmic ratio. Whereas in “large cell dysplasia” the cell diameter is at least two-fold the nuclei diameter (**Figures 1A, B**). Transgressing blood vessels and intracytoplasmic vacuoles are also considered as features of diagnostic value for identifying HCN (22, 25, 28–31) (**Figures 2A B**). “Dysplasia”, in association with the above-mentioned criteria, is diagnostic of a neoplasm than a benign nodule with extensive oxyphilic change. According to TBSRTC these lesions should be classified in the category FNHCT/SFNHCT with no comments towards adenoma or carcinoma (11).

### Tumors of Uncertain Malignant Potential (TUMPs)

Tumors of uncertain malignant potential are encapsulated neoplasms with follicular architecture and questionable capsular or vascular invasion. They are classified in two categories, based on the presence of nuclear characteristics of PTC. The follicular tumor of UMP (FT-UMP) is composed of follicular cells with no features of PTC, whereas the well differentiated tumor of UMP (WDT-UMP) shows well or partially defined nuclear features of PTC (5). FT-UMPs can also show Hürthle cell morphology (32). Considering that the diagnosis is based entirely on histological criteria, FT-UMPs consisting of Hürthle cells cannot be distinguished by cytology from HCN (HCA, HCC).

### Tumors With Low-Grade Malignant Potential

Non-invasive follicular neoplasm with papillary-like nuclear features (NIFTP) is a circumscribed tumor with no capsular invasion, showing follicular architectural pattern and PTC nuclear features. It is associated with extremely low malignant potential (5). The diagnosis of NIFTP is histological, after detailed examination of tumor capsule and the adjacent thyroid parenchyma. Oncocytic morphology is not described among the diagnostic criteria of NIFTP, according to WHO classification. However, recent publications describe cases of NIFTP showing oncocytic features. These “oncocytic” NIFTPs share the same clinical outcome and RAS-related molecular profile with the “classical” type. In addition, they bear mitochondrial DNA mutations, attributed to their oncocytic phenotype (6–8). In FNA samples they show typical Hürthle cell morphology, indistinguishable from HCN (HA, HCC) (8). NIFTP cytology befalls to the indeterminate categories AUS/FLUS, SFN, SFM (8, 11).

## Malignant Tumors

Apart from HCC other malignant tumors can show oncocytic features, such as variants of papillary, poorly differentiated, and medullary carcinoma.

### Papillary Thyroid Carcinoma (PTC)

The morphologic variants of PTC are well documented and described in the recent WHO classification (5). Recognizing PTC subtypes in cytology routine practice is occasionally challenging mainly due to unfamiliarity with rare variants. Identification of aggressive PTC subtypes preoperatively can change the design of the surgical approach and have a serious impact on the patient management and outcome (33).

Four PTC variants exhibit oncocytic cell morphology. The tall cell variant (TCV-PTC) and the hobnail variant (HV-PTC) are associated with aggressive behavior (34–37), whereas the oncocytic variant (OV-PTC) and the Warthin like variant (WL-PTC) have favorable prognosis similar to conventional PTC (38–40).

### Tall Cell Variant PTC

**Figure 3:**
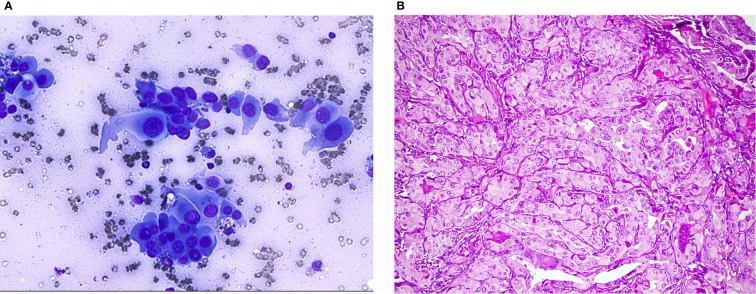
Tall cell variant of papillary thyroid carcinoma: **(A)** (Giemsa stain ×40) Neoplastic cells showing nuclear characteristics of PTC and elongated cytoplasm. **(B)** (HE stain ×20) Histological section showing elongated follicles with parallel arrangement. See the nuclear features of PTC and the eosinophilic cytoplasm with distinct cell borders.

In FNA, the TCV-PTC is easily recognized as PTC showing all the typical features, though true papillary structures are not frequent. The nuclei are enlarged hypochromatic with prominent centrally located nucleoli and frequent, often multiple nuclear pseudoinclusions occasionally giving “a soap bubble” appearance. The cytoplasm is abundant, granular with well-defined borders and the cell shape vary from polygonal, elongated, or even cylindrical with eccentric nuclei (**Figures 3A, B**). The latter are usually described as “tail-like” cells. The background may have colloid and some lymphocytes (33, 41–43). If this “tall” morphology is appreciated in many cells of the sample a possibility TC-PTC may be raised. According to TBSRTC, is classified in the category malignant, PTC with a comment that tall cell variant is favored (11).

### Hobnail Variant PTC

HV-PTC is a newly described type of PTC associated with poor prognosis (34, 37). In cytology specimens the cells are arranged in papillary-like and in micropapillary, tufting structures with prominent “hobnail” cells. There is a tendency towards loss of polarity and cohesion, therefore nuclear crowding and overlapping is not discernible. The hobnail cells have large eccentric nuclei bulging out of the dense oxyphilic cytoplasm. Also, single cells with eccentric nuclei and elongated cytoplasm described as “tear drop” cells are encountered. All PTC features are observed and “soap bubble” nuclei are also prominent (33, 36, 37). According to TBSRTC it is classified in the malignant PTC category.

TCV-PTC and HV-PTC have “aggressive looking” cells with all the PTC nuclear characteristics readily identified. Therefore, the differential diagnosis from Hürthle cell neoplasms is not problematic.

### Oncocytic Variant PTC (OV-PTC)

**Figure 4:**
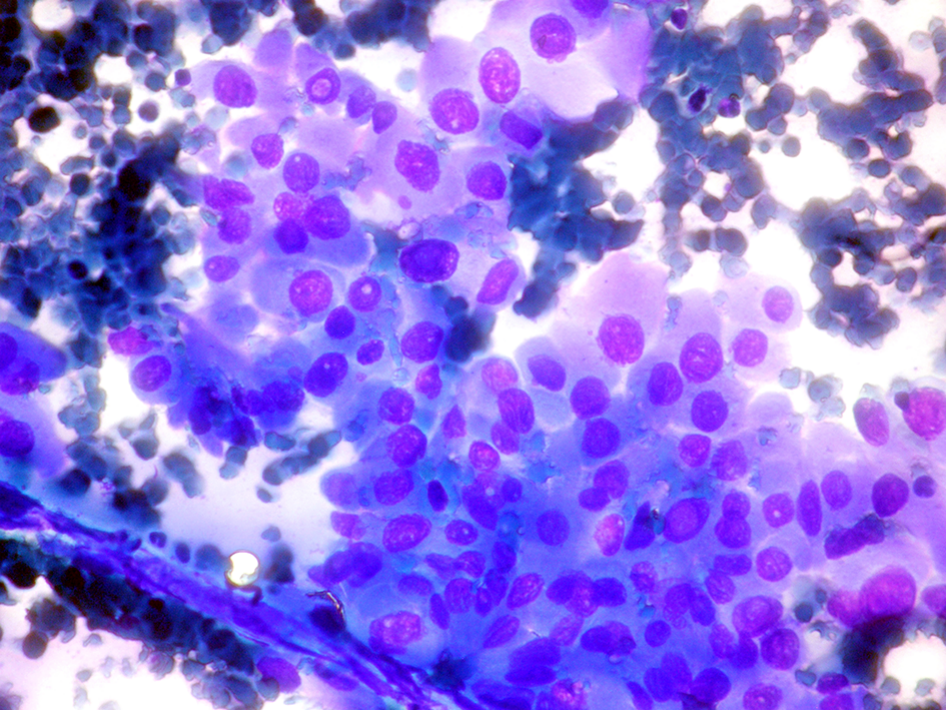
Oncocytic variant papillary thyroid carcinoma: (Giemsa stain ×40) Neoplastic cells with abundant dense cytoplasm in monolayer with minimal crowding. The cells show prominent nuclear atypia, with oval nuclei and intranuclear inclusions.

The majority of cells (>75%) in aspiration samples of OV-PTC show granular oxyphilic cytoplasm with well-defined borders. The cells are in monolayers with honeycomb like cell arrangement, microfollicles, or lie dispersed, rather than true papillary structures. In addition, OV-PTC shows central macronucleoli, in contrast to the small eccentric nucleoli, observed in classical PTC. All PTC nuclear characteristics namely enlargement, oval shape, pale chromatin, nuclear membrane irregularity, nuclear grooves, and nuclear pseudoinclusions are present (33) (**Figure 4**).

**Figure 5:**
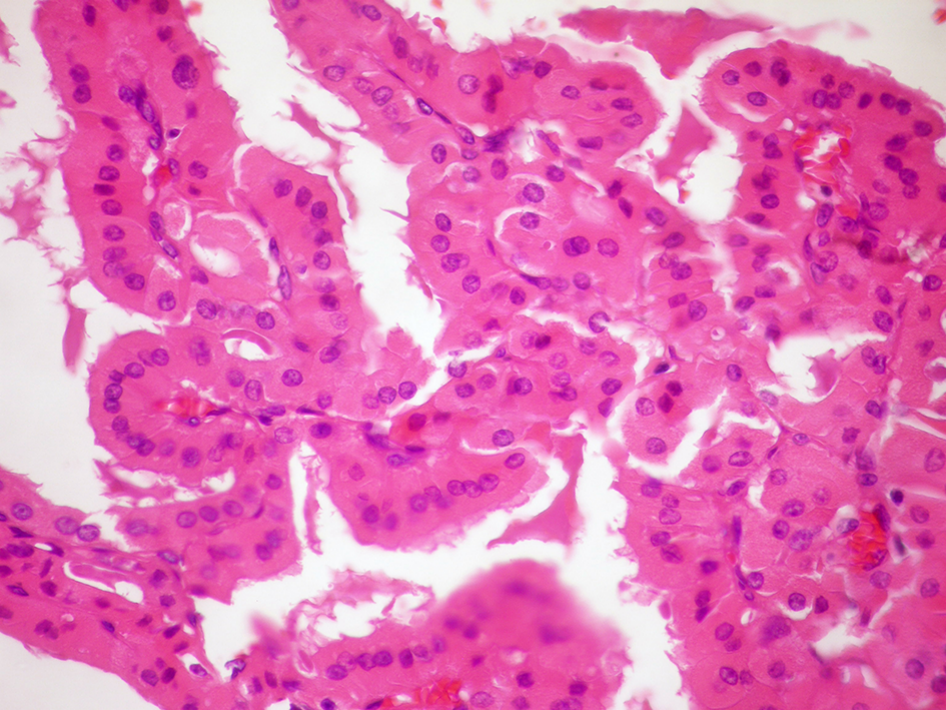
(HE stain ×40): Histology of Hürthle cell adenoma with papillary architecture.

Earlier studies have shown that 12.8 to 26% of cases considered as HCN in cytology were eventually diagnosed as PTCs on the surgical specimen. This fact highlights the difficulty to interpret correctly atypia of neoplasms with oncocytic features on cytology (44–46). Furthermore, rare HCN show focal or extensive true papillary architecture (**Figure 5**). Histological, immunohistochemical, molecular, and clinical studies have proved that these encapsulated papillary oncocytic neoplasms (EPON) are mostly related to follicular neoplasms and should be distinguished from papillary thyroid carcinoma (47, 48). Although FNA cytology of these tumors is intriguing, it can lead to the correct diagnosis (49). Papillary architecture should not be a synonym of malignancy. Searching for the presence or absence of all typical nuclear features of PTC is essential (1, 5, 33, 47–49). These cases can be classified either as category FNHCT/SFNHCT or as SFM with a note explaining the differential diagnosis (11).

### Warthin-Like Variant PTC (WLV-PTC)

WLV-PTC is a circumscribed, though not encapsulated tumor, which shows a histologic pattern reminiscent of Warthin tumor of the parotid gland, often encountered in a background of Hashimoto thyroiditis (40, 50, 51).

In cytology specimens, large polygonal tumor cells with abundant dense granular oncocytic cytoplasm are observed in papillary formation or dispersed in a lymphoplasmacytic background. The tumor cells show all the nuclear features of PTC. Lymphocytes are seen infiltrating the epithelial groups and permeating the fibrovascular cores of the papillary structures. This latter feature is helpful in distinguishing WLV-PTC from the other oncocytic PTC variants, and from non-oncocytic PTC arising in a background of Hashimoto thyroiditis (19, 40). WLV-PTC, due to the exaggerated nuclear atypia, may raise suspicion of an aggressive type as TCV-PTC or HV-PTC (33). However, in WLV-PTC there are no elongated cells, the cells have larger amount of granular cytoplasm nucleoli are more prominent, and lymphocytes are abundant (33, 40).

### Oncocytic Variant Poorly Differentiated Thyroid Carcinoma (OV-PDTC)

Poorly differentiated thyroid carcinoma (PDTC) is a rare thyroid carcinoma of follicular origin showing aggressive behavior with tendency of local recurrence, regional and distant metastases, and an overall 5-year survival of 60–70%. The oncocytic variant of PDTC (OV-PDTC) represents 30% of the cases in previous publications and is associated with even worse survival rates (52–54). Cytological diagnosis of PDTC is challenging due to the rarity of these tumors and the overlapping characteristics with follicular tumors. In previous studies including large series of PDTC only 32.5% of the cases were classified correctly with FNA (55, 56).

Cytologic criteria of PDTC are cellular smears showing insular, solid, or trabecular cell arrangement with crowding, and disperse cells. The cells are small monomorphic with high N/C ratio and variable atypia. In addition, apoptosis, mitoses are frequent. Necrosis, with debris and leukocytes in the background, may occasionally be observed. As a rule, colloid is scant or absent. Endothelial cells forming transgressing vessel or wrapped around the neoplastic blasts are also encountered (11, 55).

There is only one report in the literature where OV-PDTC was suggested preoperatively on cytology. This case met the above-mentioned diagnostic criteria of PDTC, but the cells showed abundant granular oncocytic cytoplasm (57).

It is very important to differentiate OV-PDTC from HCNs, due to its aggressive behavior which requires a radical surgical approach. Small cell size, nuclear hyperchromasia and atypia, lack of macronucleoli, crowding, and necrosis are features uncommon for HCNs. Similarly, differentiating OV-PDTC from oncocytic variant of medullary thyroid carcinoma (OV-MTC) and metastatic tumors is not easy (46, 58). MTC shows salt and pepper chromatin and may also have amyloid in the background. An essential immunohistochemical panel including thyroglobulin, thyroid transcription factor 1 (TTF1) calcitonin, and carcinoembryonic antigen (CEA) is required to establish the correct diagnosis (57, 58).

According to TBSRTC if all cytologic criteria are identified it should be classified as malignant with a short description of the features suggestive of PDTC. However, if the findings are suspicious but not conclusive for malignancy it is preferable to be classified as SFN with a description of atypical cell features, signs of necrosis, and mitotic activity (11).

### Oncocytic Variant of Medullary Thyroid Carcinoma (OV-MTC)

Medullary thyroid carcinoma (MTC) is recognized as the “great mimicker”, due to the great variety of morphological subtypes. The sensitivity of FNA in MTC diagnosis has been reported up to 89%, though a percentage of 56% has been estimated in a metanalysis study (59, 60).

OV-MTC is the only thyroid tumor with oncocytic morphology not originating from follicular cells. The diagnosis in FNA specimen is challenging even for experienced cytopathologists. The literature includes only one case of OV-MTC, diagnosed on FNA specimen, suspected by morphology and confirmed by immunohistochemistry (61). In another case molecular analysis of the aspiration material suggested the possibility of MTC in a neoplasm, otherwise diagnosed as a Hürthle cell adenoma by both cytology and histology (62).

**Figure 6:**
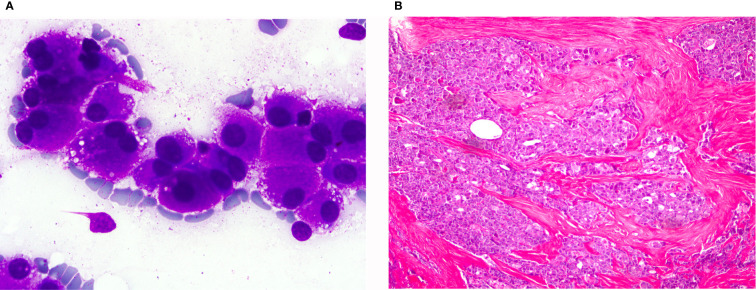
Oncocytic variant of medullary thyroid carcinoma: **(A)** (Giemsa stain ×40) Neoplastic isolated cells with abundant granular cytoplasm. **(B)** (HE ×20) Histological sections showing infiltrating groups of neoplastic cells. These cells were positive for calcitonin.

Overlapping features with HCNs lead to diagnostic pitfalls (63–65). However, it is of utmost importance to differentiate preoperatively an OV-MTC from an HCN. HCNs occasionally show disperse cell pattern reminiscent of MTC, which further complicates the differential diagnosis. One valuable clue is the quality of the cytoplasm. In HCNs it is dense and granular, whereas in OV-MTC it is loosely granular, with multivacualization similar to histiocytes (**Figures 6A, B**). In addition, if cytoplasmic metachromatic granules in Romanowsky stain are discernible the diagnosis of MTC should be considered. Generally, MTCs show more prominent nuclear atypia and pleomorphism. The typical chromatin of neuroendocrine tumors can be appreciated in careful examination of OV-MTC cells, but the presence of prominent nucleoli can be misleading. Amyloid, if present, is a very helpful feature, though it can be mistaken for colloid. Sparse nuclear inclusions and microcalcifications may be observed and mislead to the erroneous diagnosis of PTC with oncocytic morphology. If MTC is included in the differential diagnosis, immunohistochemistry should be performed and measurement of calcitonin blood levels should be considered (61, 65).

According to TBSRTC the category malignant should be used only when the cytology is conclusive, and a sub-classification of the tumor is feasible. However, if the possibility of MTC is raised but the diagnosis is not definite due to scarce material or unusual cytological features, SFM, suspicious for MTC is more appropriate. A comment recommending correlation with calcitonin serum levels or repeat aspiration for immunohistochemistry or molecular study should be included (11).

## Molecular Cytology Testing and its use in Evaluating Hürthle Cell Neoplasms

There are mainly two types of challenges in evaluating aspirates with the predominance of Hürthle cell morphology. One is to decide if it is an HCN or not, the second is if it is Hürthle cell neoplasm, then is it an HCA or HCC? Obviously, answering the second question is not compatible with real life since this challenge cannot be overcome by using cytology material without proving the presence of either vascular or capsular invasion in surgical pathology specimen (5, 11). The first challenge stems from the large spectrum of differential diagnosis of Hürthle cell neoplasms that was mentioned above in this article. There have been remarkable molecular advances in the understanding of the molecular genetics and epigenetics of HCNs in recent years (2). The characteristic alterations in oncocytic neoplasms are the increased mutations in mitochondrial DNA (mtDNA), that are distributed throughout the mitochondrial genome (tRNAs and rRNAs). This predominantly affecting genes encoding for complex I (CI) subunits of oxidative phosphorylation (OXPHOS) system, particularly *NADH-dehydrogenase 1* (ND1) and *NADH-dehydrogenase 5* (ND5) genes (2, 66). The 4977bp deletion in mtDNA which is known as “common deletion” is also frequent in HCNs of thyroid. Besides the somatic mtDNA mutations, variants of mtDNA variants also were described by Maximo et al. to be associated with HCC (2, 67). The most common gene mutations such as *rat sarcoma gene* (RAS), v*-Raf murine sarcoma viral oncogene homolog B* (BRAF), and *Telomerase reverse transcriptase promoter gene* (TERTp) mutations were described in HCC but always in lower frequency than in non-oncocytic neoplasms (2, 68–74). Similar results were found for *paired box 8/Peroxisome proliferator-activated receptor gamma gene* (PAX8/PPARg) and *rearranged during transfection gene* (RET/PTC) rearrangements (70, 75–79). Copy number variations were found more common in HCNs; Chromosomal gains were mostly seen in chromosomes 5, 7, and 12 and loss in chromosome 22 (2). Corver et al. documented “near-haploid” genotype and homozygosity in several chromosomes of the cases of HCC series, while chromosome 7 retained heterozygosity in the same group of cases, suggesting that it is important to favor the acquisition of oncocytic morphology (80, 81). Nikiforova et al. showed the overexpression of microRNAS: miR-183, miR-197, and miR-339 in HCCs and miR-31, miR-183, and miR-339 in HCAs in comparison with normal thyroid tissues together with the cluster analysis (82). These results endorsed the particular miRNA profile of HCNs and showing HCNs might be different class of tumor rather than a variant of non-oncocytic group. In 2013, Ganly et al. performed microarray analysis of gene expression in HCNs by using a clustering analysis, the authors observed that HA were more similar to MI-HCC (minimal invasive-HCC), whereas WI-HCC (widely invasive-HCC) were more distant from them with few exceptions (79). In 2018, the same group published a genomic report through RNA sequencing. The group showed MI-HCC and WI-HCC tend to cluster at some extend separately, based mostly in different enrichment in genes related to Eukaryotic Initiation Factor-2 and 4 (EIF2, EIF4) and mTOR pathways and related to mitochondrial activity (69).

Following the description of these molecular alterations, now, there are molecular tests such as the Afirma gene expression classifier (GEC) and ThyroSeq mutational panel applicable for the cytology material to stratify cytologically indeterminate thyroid nodules. Despite showing 92% sensitivity and 52% specificity for malignancy, the performance of the Afirma GEC was lower for Hürthle cell lesions (83–89). The most recent version of Afirma, the genomic sequencing classifier (GSC) uses a new algorithm incorporating RNAm expression, sequencing, and genomic copy number detection (10, 90). The most important improvements were in the categorization of Hürthle cell lesions. The sensitivity and specificity for HCN (HCA and HCC) were 89 and 59% respectively, compared with 89 and 12% in GEC (10, 90). The recent studies showed that the benign rate of Hürthle cell lesions detected in FNA is three times higher using GCS compared with GEC (91).

The latest version of ThyroSeq (ThyroSeq v3; multigene next-generation sequencing- based test) adding the copy number alterations have showed reliable performance in differentiating HCCs with the 93% sensitivity and 69% specificity (90). The study of Schatz-Siemers et al. focused a cohort of Hürthle cell lesions with indeterminate cytology by using ThyroSeq® (8). Negative results were found to be helpful for ruling out malignancy in the presurgical management of Hürthle cell lesions with indeterminate cytology (92). RAS mutations were the most prevalent and mostly they were associated with benign lesions and NIFTP (5). However, PAX8/PPARG rearrangement, *phosphatidylinositol-4,5-bisphosphate 3-kinase*, *catalytic subunit alpha* (PIK3CA), TP53, and TERT gene mutations were detected in malignant cases (2). Due to the limited data in the literature, differences in methodologies, and variability in the test version used, until this moment is difficult to evaluate the performance of ThyroSeq versions in Hürthle cell lesions or compared with non-Hürthle cell lesions observed in FNA.

## Management of HCNs

The management of HCC is similar to follicular thyroid carcinoma. There are two characteristics about the clinical behavior of HCC. One is metastatic HCC seem to be less prone to concentrating I131, and the other is more frequent locoregional lymph nodes involvement (2, 93).

The description of HCC clinical management follows the most recent American Thyroid Association guidelines and National Comprehensive Cancer Network Thyroid Cancer management guidelines (92, 93). Total thyroidectomy is indicated in cases of invasive cancer and metastatic disease, and lobectomy is indicated in minimally invasive cancer without angioinvasion. If invasive cancer is identified with vascular invasion, completion of thyroidectomy should follow. Radioactive iodine therapy should be considered in cases of gross extrathyroidal extension, when the primary tumor is more than 4 cm, when there is extensive vascular invasion, or when post- operative unstimulated thyroglobulin levels are high (94, 95). As mentioned before, HCN cannot be defined as benign or malignant based only in cytology. So, tumors suspected of being Hürthle cell cancer are often treated like follicular neoplasms. A lobectomy is usually performed at first step. If cancer is confirmed, a completion thyroidectomy needs to be done. A bilateral thyroidectomy might be considered at the first step if there are signs that the cancer has spread or if the patient wants to avoid having a second surgery afterward (11, 94, 95).

## Author Contributions

ET: drafted the manuscript. SC: conceptualization and revision. FS: conceptualization and revision. All authors contributed to the article and approved the submitted version.

## Funding

This work was partially supported by Portuguese funds through FCT—Fundação para a Ciência e a Tecnologia—in the framework of a PhD grant to SC (SFRH/BD/147650/2019).

## Conflict of Interest

The authors declare that the research was conducted in the absence of any commercial or financial relationships that could be construed as a potential conflict of interest.

## References

[1] Canberk S LiVolsi VA Baloch ZW. Oncocytic Lesions of the Neuroendocrine System. Adv Anat Pathol (2014) 21:69–82. 10.1097/PAP.0000000000000011 24508690

[2] Canberk S Lima AR Correia M Batista R Soares P Valdemar Máximo V. Oncocytic Thyroid Neoplasms: From Histology to Molecular Biology. Diagn Histopathol (2019) 25:154 – 165. 10.1016/j.mpdhp.2019.02.002

[3] Mete O Asa SL. Oncocytes, Oxyphils, Hürthle, and Askanazy Cells: Morphological and Molecular Features of Oncocytic Thyroid Nodules. Endocr Pathol (2010) 21:16–24. 10.1007/s12022-009-9102-2 20013317

[4] Montone KT Baloch ZW LiVolsi VA. The Thyroid Hürthle (Oncocytic) Cell and its Associated Pathologic Conditions: A Surgical Pathology and Cytopathology Review. Arch Pathol Lab Med (2008) 132:1241–50. 10.1043/1543-2165(2008)132[1241:TTHOCA]2.0.CO;2 18684023

[5] Lloyd RV Osamura RY Kloppel G Rosai J. WHO Classification of Tumours of Endocrine Organs. Lyon: IARC (2017).

[6] Xu B Reznik E Tuttle RM Knauf J Fagin JA Katabi N. Outcome and Molecular Characteristics of non-Invasive Encapsulated Follicular Variant of Papillary Thyroid Carcinoma With Oncocytic Features. Endocrine (2019) 64:97–108. 10.1007/s12020-019-01848-6 30689169 PMC6657696

[7] Rosario PW Mourão GF. Noninvasive Follicular Thyroid Neoplasm With Papillary-Like Nuclear Features (NIFTP): A Review for Clinicians. Endocr-Relat Cancer (2019) 26:R259–66. 10.1530/ERC-19-0048 30913533

[8] Schatz-Siemers N Brandler TC Oweity T Sun W Hernandez A Levine P. ḦRthle Cell Lesions on Thyroid Fine Needle Aspiration Cytology: Molecular and Histologic Correlation. Diagn Cytopathol (2019) 47:977–85. 10.1002/dc.24247 31293091

[9] Jalaly JB Baloch ZW. Hürthle-Cell Neoplasms of the Thyroid: An Algorithmic Approach to Pathologic Diagnosis in Light of Molecular Advances. Semin Diagn Pathol (2020) 37:234–242. 10.1053/j.semdp.2020.03.004 32444244

[10] Wong KS Trevor EA Barletta JA Krane JF. Hürthle Cell Lesions of the Thyroid: Progress Made and Challenges Remaining. Cancer Cytopathol (2021) 129:347–362. 10.1002/cncy.22375. Online ahead of print 33108684

[11] Ali SZ Cibas ES. The Bethesda System for Reporting Thyroid Cytopathology: Definitions, Criteria and Explanatory Notes. Cham, Switzerland: Springer (2018).

[12] Yazgan A Balci S Dincer N Kiyak G Tuzun D Ersoy R. Hürthle Cell Presence Alters the Distribution and Outcome of Categories in the Bethesda System for Reporting Thyroid Cytopathology. Cytopathology (2014) 25:185–9. 10.1111/cyt.12093 24024935

[13] VandenBussche CJ Adams C Ali SZ Olson MT. Cytotechnologist Performance for Screening Hürthle Cell Atypia in Indeterminate Thyroid Fine-Needle Aspirates. Acta Cytol (2015) 59:377–83. 10.1159/000441939 26606302

[14] Kini SR. Guides to Clinical Aspiration Biopsy. Thyroid (1987). New York: Igaku Shoin.

[15] Nguyen GK Ginsberg J Crockford PM Villanueva RR. Hashimoto’s Thyroiditis: Cytodiagnostic Accuracy and Pitfalls. Diagn Cytopathol (1997) 16:531–6. 10.1002/(sici)1097-0339(199706)16:6<531::aid-dc12>3.0.co;2-j 9181321

[16] Mac Donald L Yazdi HM. Fine Needle Aspiration Biopsy of Hashimoto’s Thyroiditis: Sources of Diagnostic Error. Acta Cytol (1999) 43:400–6. 10.1159/000331088 10349369

[17] Malheiros DC Canberk S Poller DN Schmitt F. Thyroid FNAC: Causes of False-Positive Results. Cytopathology (2018) 29:407–17. 10.1111/cyt.12575 29768677

[18] Yi K-I Ahn S Park DY Lee J-C Lee B-J Wang S-G. False-positive Cytopathology Results for Papillary Thyroid Carcinoma: A Trap for Thyroid Surgeons. Clin Otolaryngol (2017) 42:1153–60. 10.1111/coa.12840 28130940

[19] Canberk S Firat P Schmitt F. Pitfalls in the Cytological Assessment of Thyroid Nodules. Turk Patoloji Derg (2015) 31:18–33. 10.5146/tjpath.2015.01312 26177315

[20] Harvey AM Truong LD Mody DR. Diagnostic Pitfalls of Hashimoto’s/Lymphocytic Thyroiditis on Fine-Needle Aspirations and Strategies to Avoid Overdiagnosis. Acta Cytol (2012) 56:352–60. 10.1159/000338738 22846513

[21] Ravinsky E Safneck JR. Differentiation of Hashimoto’s Thyroiditis From Thyroid Neoplasms in Fine Needle Aspirates. Acta Cytol (1988) 32:854–61. 3201876

[22] Elliott DD Pitman MB Bloom L Faquin WC. FNA Biopsy of Hürthle Cell Lesions of the Thyroid Gland: A Cytomorphologic Study of 139 Cases With Statistical Analysis. Cancer (2006) 108:102–9. 10.1002/cncr.21716 16453320

[23] Kasper KA Stewart J Das K. Fine-Needle Aspiration Cytology of Thyroid Nodules With Hürthle Cells: Cytomorphologic Predictors for Neoplasms, Improving Diagnostic Accuracy and Overcoming Pitfalls. Acta Cytol (2014) 58:145–52. 10.1159/000358264 24525356

[24] Alaedeen DI Khiyami A McHenry CR. Fine-Needle Aspiration Biopsy Specimen With a Predominance of Hürthle Cells: A Dilemma in the Management of Nodular Thyroid Disease. Surgery (2005) 138:650–7. 10.1016/j.surg.2005.06.047 16269293

[25] Auger M. Hürthle Cells in Fine-Needle Aspirates of the Thyroid. A Review of Their Diagnostic Criteria and Significance. Cancer Cytopathol (2014) 122:241–9. 10.1002/cncy.21391 24436122

[26] Faquin WC Cibas ES Renshaw A. “Atypical” Cells in Fine-Needle Aspiration Biopsy Specimens of Benign Thyroid Cysts. Cancer (2005) 105:71–9. 10.1002/cncr.20832 15662703

[27] Yang GCH Schreiner AM Sun W. Can Abundant Colloid Exclude Oncocytic (Hürthle Cell) Carcinoma in Thyroid fine Needle Aspiration? Cytohistological Correlation of 127 Oncocytic (Hürthle Cell) Lesions. Cytopathology (2013) 24:185–93. 10.1111/j.1365-2303.2012.00988.x 22672530

[28] Renshaw A. Hürthle Cell Carcinoma is a Better Gold Standard Than Hürthle Cell Neoplasm for Fine-Needle Aspiration of the Thyroid: Defining More Consistent and Specific Cytologic Criteria. Cancer Cytopathol (2002) 96:261–6. 10.1002/cncr.10797 12378592

[29] Kini SR Miller JM Hamburger JI. Cytopathology of Hürthle Cell Lesions of the Thyroid Gland by fine Needle Aspiration. Acta Cytol (1981) 25:647–52. 6171979

[30] Yang YJ Khurana KK. Diagnostic Utility of Intracytoplasmic Lumen and Transgressing Vessels in Evaluation of Hürthle Cell Lesions by Fine-Needle Aspiration. Arch Pathol Lab Med (2001) 125:1031–5. 10.1043/0003-9985(2001)125<1031:DUOILA>2.0.CO;2 11473452

[31] Wu HH-J Clouse J Ren R. Fine-Needle Aspiration Cytology of Hürthle Cell Carcinoma of the Thyroid. Diagn Cytopathol (2008) 36:149–54. 10.1002/dc.2075067 18232004

[32] Mu N Juhlin CC Tani E Sofiadis A Reihnér E Zedenius J. High Ki-67 Index in Fine Needle Aspiration Cytology of Follicular Thyroid Tumors is Associated With Increased Risk of Carcinoma. Endocrine (2018) 61:293–302. 10.1007/s12020-018-1627-z 29796987 PMC6061212

[33] Canberk S Montezuma D Ince U Tastekin E Soares P Bongiovanni M. Variants of Papillary Thyroid Carcinoma: An Algorithmic Cytomorphology-Based Approach to Cytology Specimens. Acta Cytol (2020) 64:288–98. 10.1159/000503576 31634886

[34] Baloch Z LiVolsi VA Tondon R. Aggressive Variants of Follicular Cell Derived Thyroid Carcinoma; the So Called “Real Thyroid Carcinomas”. J Clin Pathol (2013) 66:733–43. 10.1136/jclinpath-2013-201626 23626010

[35] Silver CE Owen RP Rodrigo JP Rinaldo A Devaney KO Ferlito A. Aggressive Variants of Papillary Thyroid Carcinoma. Head Neck (2011) 33:1052–9. 10.1002/hed.21494 20824810

[36] Asioli S Maletta F Pagni F Pacchioni D Vanzati A Mariani S. Cytomorphologic and Molecular Features of Hobnail Variant of Papillary Thyroid Carcinoma: Case Series and Literature Review. Diagn Cytopathol (2014) 42:78–84. 10.1002/dc.23028 23913779

[37] Bellevicine C Cozzolino I Malapelle U Zeppa P Troncone G. Cytological and Molecular Features of Papillary Thyroid Carcinoma With Prominent Hobnail Features: A Case Report. Acta Cytol. (2012) 56(5):560–4. 10.1159/000338395 23075900

[38] Wenter V Jellinek A Unterrainer M Ahmaddy F Lehner S Albert NL. Long-Term Outcome of Rare Oncocytic Papillary (Hürthle Cell) Thyroid Carcinoma Following (Adjuvant) Initial Radioiodine Therapy. Eur J Nucl Med Mol Imaging (2019) 46:2526–35. 10.1007/s00259-019-04456-8 31410542

[39] Carr AA Yen TWF Ortiz DI Hunt BC Fareau G Massey BL. Patients With Oncocytic Variant Papillary Thyroid Carcinoma Have a Similar Prognosis to Matched Classical Papillary Thyroid Carcinoma Controls. Thyroid (2018) 28:1462–7. 10.1089/thy.2017.0603 30215297

[40] Baloch ZW LiVolsi VA. Warthin-Like Papillary Carcinoma of the Thyroid. Arch Pathol Lab Med (2000) 124:1192–5. 10.1043/0003-9985(2000)124<1192:WLPCOT>2.0.CO;2 10923082

[41] Lastra RR LiVolsi VA Baloch ZW. Aggressive Variants of Follicular Cell-Derived Thyroid Carcinomas: A Cytopathologist’s Perspective. Cancer. Cytopathol (2014) 122:484–503. 10.1002/cncy.21417 24664970

[42] Solomon A Gupta PK LiVolsi VA Baloch ZW. Distinguishing Tall Cell Variant of Papillary Thyroid Carcinoma From Usual Variant of Papillary Thyroid Carcinoma in Cytologic Specimens. Diagn Cytopathol (2002) 27:143–8. 10.1002/dc.10156 12203860

[43] Das DK Mallik MK Sharma P Sheikh ZA Mathew PA Sheikh M. Papillary Thyroid Carcinoma and its Variants in Fine Needle Aspiration Smears. A Cytomorphologic Study With Special Reference to Tall Cell Variant. Acta Cytol (2004) 48:325–36. 10.1159/000326381 15192947

[44] Pu RT Yang J Wasserman PG Bhuiya T Griffith KA Michael CW. Does Hurthle Cell Lesion/Neoplasm Predict Malignancy More Than Follicular Lesion/Neoplasm on Thyroid Fine-Needle Aspiration? Diagn Cytopathol (2006) 34:330–4. 10.1002/dc.20440 16604553

[45] Giorgadze T Rossi ED Fadda G Gupta PK Livolsi VA Baloch Z. Does the Fine-Needle Aspiration Diagnosis of “Hürthle-Cell Neoplasm/Follicular Neoplasm With Oncocytic Features” Denote Increased Risk of Malignancy? Diagn Cytopathol (2004) 31:307–12. 10.1002/dc.20132 15468114

[46] Roh MH Jo VY Stelow EB Faquin WC Zou KH Alexander EK. The Predictive Value of the Fine-Needle Aspiration Diagnosis “Suspicious for a Follicular Neoplasm, Hurthle Cell Type” in Patients With Hashimoto Thyroiditis. Am J Clin Pathol (2011) 135:139–45. 10.1309/AJCP0RW2WMDUAKGK 21173136

[47] Mai KT Elmontaser G Perkins DG Thomas J Stinson WA. Benign Hürthle Cell Adenoma With Papillary Architecture: A Benign Lesion Mimicking Oncocytic Papillary Carcinoma. Int J Surg Pathol (2005) 13:37– 41. 10.1177/106689690501300105 15735853

[48] Woodford RL Nikiforov YE Hunt JL Bellizzi AM Zhang X. Mills SE, et al. Encapsulated Papillary Oncocytic Neoplasms of the Thyroid: Morphologic, Immunohistochemical, and Molecular Analysis of 18 Cases. Am J Surg Pathol (2010) 34:1582–90. 10.1097/PAS.0b013e3181f2d820 20924280

[49] Bellevicine C Malapelle U Docimo G Ciancia G Mossetti G Pettinato G. Multicentric Encapsulated Papillary Oncocytic Neoplasm of the Thyroid: A Case Diagnosed by a Combined Cytological, Histological, Immunohistochemical, and Molecular Approach. Diagn Cytopathol (2012) 40:450–4. 10.1002/dc.21828 21965084

[50] Apel RL Asa SL LiVolsi VA. Papillary Hürthle Cell Carcinoma With Lymphocytic Stroma. “Warthin-Like Tumor” of the Thyroid. Am J Surg Pathol (1995) 19:810–4. 10.1097/00000478-199507000-00009 7793479

[51] Kim J Lim BJ Hong SW Pyo JY. Preoperative Cytologic Diagnosis of Warthin-Like Variant of Papillary Thyroid Carcinoma. J Pathol Transl Med (2018) 52:105–9. 10.4132/jptm.2017.12.26 PMC5859244 29429327

[52] Dettmer M Schmitt A Steinert H Moch H Komminoth P Perren A. Poorly Differentiated Oncocytic Thyroid Carcinoma—Diagnostic Implications and Outcome. Histopathology (2012) 60:1045–51. 10.1111/j.1365-2559.2012.04188.x53 22348590

[53] Asioli S Erickson LA Righi A Jin L Volante M Jenkins S. Poorly Differentiated Carcinoma of the Thyroid: Validation of the Turin Proposal and Analysis of IMP3 Expression. Mod Pathol (2010) 23:1269–78. 10.1038/modpathol.2010.117 20562850

[54] Wong KS Lorch JH Alexander EK Marqusee E Cho NL Nehs MA. Prognostic Significance of Extent of Invasion in Poorly Differentiated Thyroid Carcinoma. Thyroid (2019) 29:1255–61. 10.1089/thy.2019.0263 31397224

[55] Bongiovanni M Bloom L Krane JF Baloch ZW Powers CN Hintermann S. Cytomorphologic Features of Poorly Differentiated Thyroid Carcinoma: A Multi-Institutional Analysis of 40 Cases. Cancer Cytopathol (2009) 117:185–94. 10.1002/cncy.20023 19365842

[56] Kane SV Sharma TP. Cytologic Diagnostic Approach to Poorly Differentiated Thyroid Carcinoma: A Single-Institution Study. Cancer Cytopathol (2015) 123:82–91. 10.1002/cncy.21500 25557073

[57] Onenerk M Canberk S Gunes P Erkan M Kilicoglu GZ. Oncocytic Variant of Poorly Differentiated Thyroid Carcinoma: “Is Diagnosis Possible by Fine-Needle Aspiration?” Cytojournal (2016) 13:23. 10.4103/1742-6413.192188 27761148 PMC5070041

[58] Laforga JB Cortés VA. Oncocytic Poorly Differentiated (Insular) Thyroid Carcinoma Mimicking Metastatic Adenocarcinoma. A Case Report and Review of the Literature. Diagn Cytopathol (2019) 47:584–8. 10.1002/dc.24147 30637975

[59] Papaparaskeva K Nagel H Droese M. Cytologic Diagnosis of Medullary Carcinoma of the Thyroid Gland. Diagn Cytopathol (2000) 22:351–8. 10.1002/(sici)1097-0339(200006)22:6<351::aid-dc5>3.0.co;2-t 10820528

[60] Trimboli P Treglia G Guidobaldi L Romanelli F Nigri G Valabrega S. Detection Rate of FNA Cytology in Medullary Thyroid Carcinoma: A Meta-Analysis. Clin Endocrinol (2015) 82:280–5. 10.1111/cen.12563 25047365

[61] Canberk S Onenerk M Gunes P Sayman E Kilicoglu G. Oncocytic Variant of Medullary Thyroid Carcinoma. Endocr Pathol (2015) 26:320–3. 10.1007/s12022-015-9389-0 26293669

[62] Spaulding SL Ho R Everest S Chai RL. The Role of Molecular Testing in the Diagnosis of Medullary Thyroid Cancer: A Case Report of Oncocytic Medullary Thyroid Carcinoma and Review of the Literature. Am J Otolaryngol (2020) 41:102312. 10.1016/j.amjoto.2019.102312 31727331

[63] Dedivitis RA Di Giovanni JH Silva GF Marinho LC Guimarães AV. Oncocytic Variant Ofmedullary Thyroid Carcinoma: Case Report. Arq Bras Endocrinol Metabol (2004) 48:315–7. 10.1590/s0004-27302004000200017 15640889

[64] Kaushal S Iyer VK Mathur SR Ray R. Fine Needle Aspiration Cytology of Medullary Carcinoma of the Thyroid With a Focus on Rare Variants: A Review of 78 Cases. Cytopathology (2011) 22:95–105. 10.1111/j.1365-2303.2010.00747.x 20518799

[65] Sams SB Tompkins KD Mayson S Raeburn CD Mehrotra S. Oncocytic Variant of Medullary Thyroid Carcinoma; A Rare Tumor With Numerous Diagnostic Mimics by Fine Needle Aspiration. Diagn Cytopathol (2017) 45:1148–52. 10.1002/dc.23790 28802094

[66] Maximo V Sobrinho-Simoes M. Hürthle Cell Tumours of the Thyroid. A Review With Emphasis on Mitochondrial Abnormalities With Clinical Relevance. Virchows Arch (2000) 437:107–15. 10.1007/s004280000219 10993269

[67] Maximo V Sobrinho-Simoes M. Mitochondrial DNA ‘Common’ Deletion in Hürthle Cell Lesions of the Thyroid. J Pathol (2000) 192:561–2. 10.1002/1096-9896(200012)192:4<561::AID-PATH790>3.0.CO;2-3 11113879

[68] Ganly I McFadden DG. Short Review: Genomic Alterations in Hürthle Cell Carcinoma. Thyroid (2019) 29:471–9. 10.1089/thy.2019.0088 30848171

[69] Ganly I Makarov V Deraje S Dong Y Reznik E Seshan V. Integrated Genomic Analysis of Hürthle Cell Cancer Reveals Oncogenic Drivers, Recurrent Mitochondrial Mutations, and Unique Chromosomal Landscapes. Cancer Cell (2018) 34:256–70. 10.1016/j.ccell.2018.07.002 PMC6247912 30107176

[70] Nikiforova MN Lynch RA Biddinger PW Alexander EK Dorn GW Tallini G. RAS Point Mutations and PAX8-PPAR Gamma Rearrangement in Thyroid Tumors: Evidence for Distinct Molecular Pathways in Thyroid Follicular Carcinoma. J Clin Endocrinol Metab (2003) 88:2318–26. 10.1210/jc.2002-021907 12727991

[71] Melo M da Rocha AG Vinagre J Batista R Peixoto J Tavares C. TERT Promoter Mutations are a Major Indicator of Poor Outcome in Differentiated Thyroid Carcinomas. J Clin Endocrinol Metab (2014) 99:E754–765. 10.1210/jc.2013-3734 PMC4191548 24476079

[72] Chindris AM Casler JD Bernet VJ Rivera M Thomas C Kachergus JM. Clinical and Molecular Features of Hürthle Cell Carcinoma of the Thyroid. J ClinEndocrinol Metab (2015) 100:55–62. 10.1210/jc.2014-1634 25259908

[73] Vinagre J Almeida A Pópulo H Batista R Lyra J Pinto V. Frequency of TERT Pro- Moter Mutations in Human Cancers. Nat Commun (2013) 4:2185. 10.1038/ncomms3185 23887589

[74] Landa I Ganly I Chan TA Mitsutake N Matsuse M Ibrahimpasic T. Frequent Somatic TERT Promoter Mutations in Thyroid Cancer: Higher Prevalence in Advanced Forms of the Disease Frequent Somatic TERT Promoter Mutations in Thyroid Cancer: Higher Prevalence in Advanced Forms of the Disease. J Clin Endocrinol Metab (2013) 98:E1562–1566. 10.1210/jc.2013-2383 PMC3763971 23833040

[75] de Vries MM Celestino R Castro P Eloy C Máximo V Van Der Wal JE. RET/PTC Rearrangement is Prevalent in Follicular Hürthle Cell Carcinomas. Histopathology (2012) 61:833–43. 10.1111/j.1365-2559.2012.04276.x 22803838

[76] Cheung CC Ezzat S Ramyar L Freeman JL Asa SL. Molecular Basis Off Hürthle Cell Papillary Thyroid Carcinoma. J Clin Endocrinol Metab (2000) 85:878–82. 10.1210/jcem.85.2.6404 10690905

[77] Chiappetta G Toti P Cetta F Giuliano A Pentimalli F Amendola I. The RET/PTC Oncogene is Frequently Activated in Oncocytic Thyroid Tumors (Hürthle Cell Adenomas and Carcinomas), But Not in Oncocytic Hyperplastic Lesions. J Clin Endocrinol Metab (2002) 87:364–49. 10.1210/jcem.87.1.8180 11788677

[78] Gopal RK Kübler K Calvo SE Polak P Livitz D Rosebrocket D. Widespread Chromosomal Losses and Mitochondrial DNA Alterations as Genetic Drivers in Hürthle Cell Carcinoma. Cancer Cell (2018) 34:242–355. 10.1016/j.ccell.2018.06.013 30107175 PMC6121811

[79] Ganly I Ricarte Filho J Eng S Ghossein R Morris LG Liang Y. Genomic Dissection of Hürthle Cell Carcinoma Reveals a Unique Class of Thyroid Malignancy. J Clin Endocrinol Metab (2013) 98:E962–972. 10.1210/jc.2012-3539 PMC5393465 23543667

[80] Corver WE Ruano D Weijers K den Hartog WC van Nieuwenhuizen MP de Miranda N. Genome Haploidisation With Chromosome 7 Retention in Oncocytic Follicular Thyroid Carcinoma. PloS One (2012) . 7:e38287. 10.1371/journal.pone.0038287 22675538 PMC3365880

[81] Corver WE van Wezel T Molenaar K Schrumpf M van den Akker B van Eijk R. Near-haploidization Significantly Associates With Oncocytic Adrenocortical, Thyroid, and Parathyroid Tumors But Not With Mitochondrial DNA Mutations. Genes Chromosomes Cancer (2014) 53:833–44. 10.1002/gcc.22194 24909752

[82] Nikiforova MN Tseng GC Steward D Diorio D Nikiforov YE. MicroRNA Expression Profiling of Thyroid Tumors: Biological Significance and Diagnostic Utility. J Clin Endocrinol Metab (2008) 93:1600–8. 10.1210/jc.2007-2696 PMC2386678 18270258

[83] Brauner E Holmes BJ Krane JF Nishino M Zurakowski D Hennessey JV. Performance of the Afirma Gene Expression Classifier in Hürthle Cell Thyroid Nodules Differs From Other Indeterminate Thyroid Nodules. Thyroid (2015) 25:789–96. 10.1089/thy.2015.0049 25962906

[84] Lastra RR Pramick MR Crammer CJ LiVolsi VA Baloch ZW. Implications of a Suspicious Afirma Test Result in Thyroid Fine-Needle Aspiration Cytology: An Institutional Experience. Cancer Cytopathol (2014) 122:737–44. 10.1002/cncy.21455 25123499

[85] Yang SE Sullivan PS Zhang J Govind R Levin MR Rao JY. Has Afirma Gene Expression Classifier Testing Refined the Indeterminate Thyroid Category in Cytology? Cancer Cytopathol (2016) 124:100–9. 10.1002/cncy.21624 26422098

[86] McIver B Castro MR Morris JC Bernet V Smallridge R Henry M. An Independent Study of a Gene Expression Classifier (Afirma) in the Evaluation of Cytologically Indeterminate Thyroid Nodules. J Clin Endocrinol Metab (2014) 99:4069–77. 10.1210/jc.2013-3584 24780044

[87] Alexander EK Kennedy GC Baloch ZW Cibas ES Chudova D Diggans J. Preoperative Diagnosis of Benign Thyroid Nodules With Indeterminate Cytology. N Engl J Med (2012) 367:705–15. 10.1056/NEJMoa1203208 22731672

[88] Harrell RM Bimston DN. Surgical Utility of Afirma: Effects of High Cancer Prevalence and Oncocytic Cell Types in Patients With Indeterminate Thyroid Cytology. Endocr Pract (2014) 20:364–9. 10.4158/EP13330.OR 24246351

[89] Wu JX Young S Hung ML Li N Yang SE Cheung DS. Clinical Factors Influencing the Performance of Gene Expression Classifier Testing in Indeterminate Thyroid Nodules. Thyroid (2016) 26:916–22. 10.1089/thy.2015.0505 27161519

[90] Patel KN Angell TE Babiarz J Barth NM Blevins T Duh QY. Performance of a Genomic Sequencing Classifier for the Preoperative Diagnosis of Cytologically Indeterminate Thyroid Nodules. JAMA Surg (2018) 153:817–24. 10.1001/jamasurg.2018.1153 PMC6583881 29799911

[91] San Martin VT Lawrence L Bena J Madhun NZ Berber E Elsheikh TM. Real-World Comparison of Afirma GEC and GSC for the Assessment of Cytologically Indeterminate Thyroid Nodules. J Clin Endocrinol Metab (2020) 105:428–35. 10.1210/clinem/dgz099 31665322

[92] Pearlstein S Lahouti AH Opher E Nikiforov YE Kuriloff DB. Thyroseq V3 Molecular Profiling for Tailoring the Surgical Management of Hürthle Cell Neoplasms. Case Rep Endocrinol (2018) 2018:9329035. 10.1155/2018/9329035 30105107 PMC6076910

[93] Ahmadi S Stang M Jiang XS Sosa JA. ḦRthle Cell Carcinoma: Current Perspectives. Onco Targets Ther (2016) 9:6873–84. 10.2147/OTT.S119980 PMC5106236 27853381

[94] Hassan A Khalid M Riaz S Nawaz MK Bashir H. Follicular Thyroid Carcinoma: Disease Response Evaluation Using American Thyroid Association Risk Assessment Guidelines. Eur Thyroid J (2015) 4:260–65. 10.1159/000442237 PMC4716413 26835430

[95] National Comprehensive Cancer Network. Thyroid Cancer (Version 1.2018) . Available at: https://www.nccn.org/professionals/physician_gls/pdf/thyroid.pdf (Accessed November 8, 2018).

